# Using Life Cycle Assessments to Measure the Environmental Impact of Alternative Care Models in the Neonatal Intensive Care Unit

**DOI:** 10.3390/ijerph23050681

**Published:** 2026-05-20

**Authors:** Thomas Walsh, Samantha House, Emily Monroe, Will Clendenning, Chad Klaas, Samantha Melgar, Ismael Rosales-Albarran, Tyler Hartman, Kathryn Richards

**Affiliations:** 1 Dartmouth Health Children’s, Lebanon, NH 03766, USA; 2 Geisel School of Medicine, Dartmouth College, Hanover, NH 03755, USA; 3 Thayer School of Engineering, Dartmouth College, Hanover, NH 03755, USA

**Keywords:** neonatal intensive care unit (NICU), healthcare, carbon emissions, carbon footprint, sustainability, life cycle assessment (LCA), interdisciplinary team, clinician engineer collaboration

## Abstract

**Highlights:**

**Public health relevance—How does this work relate to a public health issue?**

- Environmental degradation and climate change are major contributors to human morbidity and mortality.
- The healthcare sector is a major contributor to global greenhouse gas emissions. Quantifying and improving the carbon footprint of the healthcare sector could have a drastic impact on environmental health and public health.

**Public health significance—Why is this work of significance to public health?**

- Reducing the healthcare sector’s carbon footprint will lead to improved environmental conditions and therefore improved health outcomes.
- Improvements in environmental conditions will have population-wide health impacts.

**Public health implications—What are the key implications or messages for practitioners, policymakers and/or researchers in public health?**

- Data on the carbon footprint of specific clinical practices are limited. More granular emissions data are key to identifying and modifying processes that generate significant emissions.
- This analysis demonstrates the potential environmental benefits of a home hospital program for neonates, which showed particular reductions in carbon footprint through reduced travel distances and waste generation.

**Abstract:**

The healthcare sector is a major contributor to global greenhouse gas emissions. Little is known about the impact of individual clinical practices on overall emissions; more granular healthcare emissions data are needed to identify opportunities for resource stewardship. Our objective was to deploy an interdisciplinary team to perform Life Cycle Assessments (LCAs) comparing carbon emissions attributable to a novel home-care program for premature infants to those attributable to routine care in the Neonatal Intensive Care Unit (NICU). We used LCA methodology to compare the carbon footprint of two weeks of traditional care of infants in our NICU to that of those enrolled in an institutional alternative care program known as “Hope Grows at Home,” which transitions eligible infants requiring nasogastric feeds to the home setting with ongoing NICU team support. Our analysis showed that in-home care produces 77 kg of CO_2_ emissions (kgCO_2_e) per infant over a 14-day period, as compared to in-hospital care, which produced 338 kgCO_2_e. Transportation to a healthcare facility accounted for the majority of emissions in both groups (292 kgCO_2_e for NICU care and 58 kgCO_2_e for home care). This finding is likely impacted by our facility’s rural location. Home care reduced solid waste emissions by approximately 94% relative to NICU care (1.74 vs. 26.97 kgCO_2_e per term), reflecting the home setting’s reuse of feeding syringes and bottles that are routinely single-use in the hospital. Prospective data collection strategies for infants enrolled in home care will further refine our results. Exploring additional interdisciplinary collaborations may facilitate similar analyses, offering more insight into environmental stewardship opportunities within healthcare.

## 1. Introduction

It is well established that the healthcare sector has a profound impact on the environment [1,2]. The U.S. healthcare sector accounts for approximately 8.5% of national greenhouse gas emissions, reaching 1692 kgCO_2_e per capita in 2018—the highest rate among industrialized nations [3,4]. Current literature has established that healthcare with high resource utilization, such as intensive care units, can generate more than double the emissions compared to lower levels of care [5,6]. Although this example represents a growing understanding of the carbon intensity of some specific clinical services, there remains a wide swath of healthcare services and pathways that have poorly characterized carbon footprints.

With this understanding, environmental stewardship has become an increasing area of focus in healthcare delivery systems. In pediatrics, the impact of climate change on children’s health has brought increasing attention to this topic [7,8,9]. However, environmental stewardship efforts among children’s hospitals remain in their infancy. One recent analysis identified that <15% of children’s hospitals are publicly tracking and reporting at least one suggested mitigation metric [10]. This gap between appreciating healthcare’s significant carbon footprint and improving the sustainability of healthcare practices motivates targeted, service-level environmental analyses of the kind reported here. The analyses highlighted in this report were the first of their kind performed at our facility, representing an effort to close the gap on sustainability within our own healthcare system.

One of the primary obstacles to assessing the environmental impact of healthcare practices is in reliable measurement and reporting of data [11,12]. In most instances the healthcare sector has borrowed existing methodologies from other industries to calculate carbon footprints. To date, efforts to quantify the carbon footprint of healthcare typically target high-level estimates, focusing on the breadth of healthcare and general utilization practices [1]. These existing efforts are largely reliant on Economic Input–Output (EEIO) analysis to determine carbon footprints [13]. In these analyses, a conversion factor is used to translate dollar amounts directly into an emissions value [13]. These conversion factors are usually sourced from governing bodies or environmental organizations (such as the EPA) [14]. While the output of these analyses can be helpful in understanding system-level impact, such methodology is not precise enough to facilitate understanding of the environmental impact of specific clinical practices [15].

In order to obtain data on specific clinical practices, the healthcare sector must utilize an alternate and more specific methodology known as Life Cycle Assessments (LCAs). LCAs are “cradle to grave” analyses that attempt to quantify all the emissions associated with the lifespan of a specific product or process [16]. These studies determine which specific elements of a process render the greatest impact on emissions. The number of LCAs performed in the healthcare sector has increased in the past five years; however, these analyses remain limited [17,18,19,20,21]. The granular data resulting from these LCAs may drive change within the healthcare system. For example, solid waste generation and its implications within the healthcare system has been previously assessed [22,23,24]. It is established that healthcare systems in high-income nations rely substantially on linear supply chains composed of single-use disposable medical devices, contributing to escalating clinical waste volumes and supply-chain emissions [25]. Service-level LCAs are foundational to this transformation, providing the precise data required to identify where single-use practices generate disproportionate environmental impact relative to clinical benefit. Additionally, there is the potential for service-level LCA data to support the development of environmental impact as a new domain of quality evaluation of clinical programs and services.

At our institution, a novel program was developed in 2020 to facilitate the transition of care for some appropriate neonates in the Neonatal Intensive Care Unit (NICU) to the home setting despite an ongoing need for medical equipment. Known as “Hope Grows at Home” (HGaH), this program has been in continuous operation since its inception, enrolling 150 patients and demonstrating positive clinical outcomes [26]. Conceptually, the HGaH program represented an alternative, service-level healthcare practice that could be compared to an existing, resource-intensive healthcare standard (care in the NICU). We therefore asked: Does this structured, home-based care program produce lower greenhouse gas emissions than equivalent NICU care, and which inventory categories drive any observed differences? We hypothesized that home-based care would yield a lower carbon footprint. To answer this question, we convened an interdisciplinary clinician–engineer team to perform a comprehensive LCA of the HGaH program against routine NICU care, using ISO 14040/44 methodology [27,28]. Secondary to testing our hypothesis, we also convened this interdisciplinary team with the aim of increasing cross-campus collaboration at our academic medical center that might support future LCA work.

## 2. Materials and Methods

### 2.1. Study Population

The HGaH program offers enrollment to premature infants between 35 and 39 weeks post-menstrual age requiring no respiratory support who remain admitted primarily for nasogastric feeding. To be eligible, infants must have completed an apnea countdown (if applicable), demonstrated stable thermoregulation out of an isolette for 48 h, and be taking >20% of feeds by mouth. Specific exclusion criteria include transfer from outside facilities, central venous access, serious congenital anomalies, chromosomal anomalies, and child protective services custody. Patients are considered on a case-by-case basis with these criteria as a guide. Parents (and/or guardians) have final determination on joining the proposed program.

### 2.2. Study Team

To achieve our aims, we first established an interdisciplinary team. Three physicians from our Pediatric Department interested in the intersection of healthcare quality and environmental health (T.W., third-year pediatric resident; T.H., attending Neonatologist; and S.A.H., attending Pediatric Hospitalist and Quality and Safety lead) developed a project outline and approached professors (E.M.) with known involvement in climate-related research at our academically affiliated school of engineering to consider collaboration opportunities. Engineering faculty proposed collaboration via a senior-level engineering course in which student teams select project proposals from outside organizations around which they are interested in developing solutions. Our clinicians crafted a formal proposal to perform an LCA on the HGaH program. As part of this proposal, the student team would be able to perform “in the field” observations of NICU care, therefore creating an opportunity to simultaneously learn about healthcare practices and LCA processes. This proposal was subsequently chosen by one of the student teams. The accepting team consisted of 4 senior-level engineering students (C.K., I.R.-A., S.M., W.C.), who were advised by industry experts in the fields of sustainability, environmental engineering and analysis. This course spanned two academic quarters (approximately six months).

### 2.3. Team Engagement and Communication

After the establishment of our team, student members led LCA performance with input from our clinician team and their faculty advisors. We held bi-weekly meetings throughout the duration of the course; meetings were augmented by regular communication via email and messaging.

In order to gain familiarity with the relevant clinical environment, the student team made multiple site visits to tour our NICU. During these visits the students were able to observe bedside care and unit processes, interview nursing staff, and perform inventory on materials and devices to inform LCA performance.

### 2.4. LCA Methodology

LCAs were conducted on two distinct patient populations: (1) neonates receiving traditional care in the NICU throughout the duration of their equipment requirements and (2) infants receiving care through the HGaH program. The infant care that was performed in the NICU was matched as closely as possible with infants who were eligible for HGaH programming (i.e., infants who met the criteria but whose families opted out of the optional program).

We developed our LCA model and analysis by following guidelines set out in the International Organization for Standardization (ISO) standards for LCAs, specifically ISO 14040 and 14044 [27,28]. In coordination with these guidelines, our LCAs were performed with a four-part methodology: goal and scope definition, inventory analysis, impact assessment, and interpretation of results. Our inventorying processes included direct, in-field observations. These direct observations were carried out with a two-fold objective: to obtain the most granular data possible on the healthcare practices being assessed, and so all members of the interdisciplinary team would have full comprehension of the processes being analyzed. Inventory and impact-assessment calculations were performed in spreadsheet format (Microsoft Excel) using emission factors from the public sources detailed in Appendix C; commercial LCA software with proprietary life cycle inventory databases was not used. This approach is consistent with comparable peer-reviewed home-care LCAs and is appropriate given the project scope and the absence of a U.S. healthcare-specific life cycle inventory database [29].

#### 2.4.1. Goal and Scope

**Figure 1:**
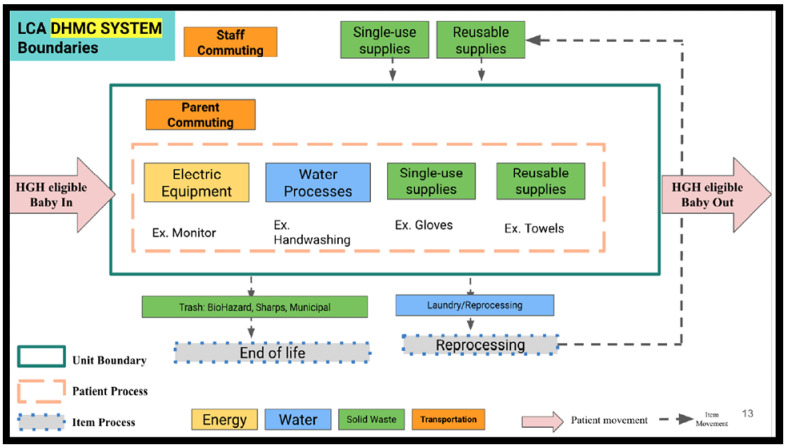
System boundary flow diagram utilized in this analysis (here the “DHMC System” is Dartmouth Hitchcock Medical Center, the hospital in which our NICU resides). This diagram depicts the inputs and outputs of the identified system, caring for an infant, and defines the system boundaries with those inputs and outputs. Everything within the unit boundary is considered part of this LCA, while everything outside of the system is not.

Setting a goal and a scope for an LCA creates a specific framework for analysis. This framework is defined by the functional unit to be studied and by developing system boundaries. The functional unit acts as a common denominator between LCAs. For this study our functional unit was defined as kilograms of CO_2_ emissions per infant. Because the average duration of supplemental feeding in the HGaH program was 14 days, we used this as the time bounds of our functional unit. Our system boundaries, which define the extent of processes to be included in the LCA, were defined to capture four fundamental categories for the scope of care of the infants in both groups: energy, water, solid waste, and transportation. System boundaries are further demonstrated in Figure 1 and Table 1.

#### 2.4.2. Inventory Analysis

Inventory analysis was completed by touring and inventorying NICU facilities, direct observation of infant care, interviewing nurses and clinical staff (Appendix A), carrying out a literature review, and referring to equipment manuals and specifications. Detailed inventory was completed by category, as briefly explained in Table 1 (complete inventory processing can be found in Appendix B). An additional component in the inventory process of this LCA was in the inclusion of “newborn cares” frequency. “Cares” are defined as the regular activities performed in the care of the newborn. “Cares” are performed in the NICU every 3 h during the 24 h day, resulting in 8 instances of cares per day. This resulted in all inventory assessments utilizing an 8×/day factor in their quantifications.

**Table 1:** Inventory analysis by impact category.

Impact Category	Included	Examples
**Energy**	Energy inventory included all direct care items with a connection to an outlet/power source.	Vital sign monitor Milk warmer
**Water**	Water inventory assessed all care activities that required water usage.	Handwashing Bathing Formula mixing
**Solid waste**	Solid waste inventory included all single-use items that are used in the care of an infant.	Diapers Gloves Feeding syringes
**Transportation**	Transportation inventory included caregiver transportation to and from the hospital during a neonate’s time in either the HGaH program or in the NICU after they reach HGaH eligibility.	Distance traveled Number of visits Transportation via a standard passenger vehicle

#### 2.4.3. Impact Assessment

Impact assessment is the process through which the environmental impact of inventory is determined. In this analysis the inventory of each impact category was converted into the chosen impact value, kilograms of carbon dioxide emissions (kgCO_2_e). These calculations are reliant on conversion factors, which are primarily created by governmental agencies and academic institutions (Appendix C, [14,30,31,32,33,34,35,36,37]). Impact-assessment calculations were performed in spreadsheet format (Microsoft Excel); commercial LCA software with proprietary life cycle inventory databases was not used. This approach is consistent with comparable peer-reviewed home-care LCAs [29]. An example of a specific impact assessment is provided in Table 2 (all other impact assessment data and calculations may be found in Appendix B). After completing impact assessments on the individual components of an impact category (e.g., all the components that constituted “solid waste”), the totals were summed to create cumulative impact values. We utilized both high and low estimates in our calculations. In our final interpretation of results, we averaged these values to obtain a final emissions value.

This table represents the impact assessment for syringes used in nasogastric feeds of NICU newborns. Syringes were collected from the hospital and weighed in order to obtain an average weight (0.025 kg). We utilized conversion factors for both material use and waste disposal in forming a cumulative emissions factor (Appendix C). Emissions per day were then calculated by multiplying the weight per day by the conversion factor. In order to account for the entirety of our functional unit, daily emissions were multiplied by 14 (the duration of HGaH enrollment in days).

#### 2.4.4. Interpretation

Interpretation of impacts was completed by comparing the carbon emissions attributed to routine NICU care vs. the HGaH program by domain. As the interpretation component of our LCA is largely synonymous with results, the interpretation will be covered in the results portion of this article.

## 3. Results

In this LCA, care provided in the NICU produced an average of 338.19 kg of CO_2_e/eligible baby over the 14-day time period. The breakdown by impact category revealed that energy accounted for 18.91 kg of CO_2_e (5.59%), water accounted for 0.31 kg of CO_2_e (0.09%), solid waste accounted for 26.97 kg of CO_2_e (7.97%), and transportation accounted for 292 kg of CO_2_e (86.34%). Care in the HGaH program produced an average of 77.15 kg of CO_2_e emissions over a 14-day period. Inspecting the breakdown by scope revealed that energy accounted for 16.34 kg of CO_2_e (21.18%), water accounted for 0.66 kg of CO_2_e (0.86%), solid waste accounted for 1.74 kg of CO_2_e (2.26%), and transportation accounted for 58.4 kg of CO_2_e (75.71%). These results are further displayed in Table 3.

Solid waste generated in the NICU amounted to a total of 15.11 kg per term. The HGaH program generated 5.97 kg of solid waste per term. Solid waste in this analysis was characterized by item type and weight rather than by regulated waste stream (regulated medical waste, sharps, municipal solid waste), and disposal pathway emissions (incineration, autoclave sterilization, landfill) were not modeled separately.

## 4. Discussion

This LCA of a novel home-care program for neonates identified that routine NICU care produced more than four-fold the carbon emissions of supported home care. For both cohorts, transportation was the major contributor to CO_2_ emissions, likely impacted by our rural location. The dominance of transportation in both care models is consistent with current state-of-the-science reviews, which identified travel, facilities, and consumables as the principal carbon contributors across 151 hospital services and care pathways spanning multiple medical specialties [38]. Per-term transport savings observed in this study (approximately 234 kgCO_2_e) fall within the 0.70–372 kgCO_2_e per-consultation range reported across telemedicine LCAs [39], suggesting the magnitude of benefit is consistent with comparable home-care and remote-care interventions despite differences in clinical context.

Travel demands can be a limiting factor in a family’s ability to regularly visit, and care for, preterm newborns in a NICU setting [40]. Without this regular interaction, infants may be missing a foundational component of newborn care [41]. This presents an obvious challenge for families and their newborns. In addition to these clinical and social concerns, our data support the notion that there is an environmental impact here as well. One of the primary impressions from this analysis is the potential environmental benefit of remote patient care, in the form of telehealth and/or remote patient monitoring. In cases where clinical outcomes can remain constant [42,43,44], a care-at-home model may represent a mode of healthcare delivery that is healthy for patients, fiscally responsible, and good for the planet.

In addition to our transportation findings, there was a notable difference in the carbon footprint of solid waste for the two care environments. This is likely attributable to the NICU’s practice of disposing plastic bottles and syringes with each use, while the home setting allows for reuse of supplies. Given that hospitals must uphold high standards of infection prevention, and therefore utilize many single-use materials and processes, this was not a particularly surprising finding. However, there are some well-known examples of healthcare delivery decreasing its waste output while maintaining care outcomes (such as the NHS’s “Gloves Off” campaign) [45]. Our LCAs highlight that there are further opportunities for the healthcare sector to examine, and potentially alter, longstanding single-use practices. Concrete waste-reduction strategies that could be evaluated for the NICU setting include extending reuse protocols for feeding syringes and milk-warmer liners where infection-prevention standards allow, reviewing single-use device decisions through a circular-economy lens in collaboration with infection-prevention and supply-chain leadership, and evaluating reprocessable alternatives for current single-use items [23,25].

An impactful aspect of this work is that these analyses were performed on significantly different levels of healthcare service provided. We do not know of a specific, comprehensive analysis of NICU care, but by extrapolating findings from other intensive care units we postulate that NICU care is similarly resource intensive [5,6,38]. There is some literature to suggest greener NICU infrastructure, but little to none on greener NICU services and pathways [46]. The HGaH program offers an alternative to the resource-intensive care in the NICU. Therefore, the HGaH program represents a creative solution in which standards of care can be maintained while significantly reducing the carbon intensity. This supports the continued re-evaluation of care practices and development of alternative care models.

Taking a broader perspective, performing this analysis demonstrated the necessity of developing and utilizing an interdisciplinary study team. One of the secondary aims of this project was to increase interdisciplinary collaboration across our academic campus. We found that the creation of our interdisciplinary team was paramount to the completion of this novel analysis at our facility. Performing these LCAs was a robust educational opportunity for our team, and the knowledge gained via our collaboration was a highly valued outcome by all involved. Our hope is that these initial LCAs will provide a framework for our institution to perform further, and more refined, analyses, with the ultimate objective being that continued collaboration may lead to more sustainable practices at our healthcare facility. Overall, this experience supports what other recent publications have highlighted: the importance of creating interdisciplinary sustainability teams [10,21]. By fully leveraging the interdisciplinary capacities of an academic medical center, establishing sustainability teams creates a robust methodology to advance sustainability initiatives across the healthcare sector.

Several limitations should be noted. First, our carbon footprint estimates were informed by direct observation, nursing interviews, and assumptions about routine care patterns rather than prospectively captured patient-level activity logs. The precision of these estimates is therefore constrained by the absence of per-patient daily data across the 14-day functional unit.

Second, while many healthcare LCAs apply commercial software paired with proprietary life cycle inventory databases (e.g., SimaPro with ecoinvent, GaBi, OpenLCA), this analysis used a process-based inventory paired with publicly available emission factors from governmental and peer-reviewed sources, including the U.S. Environmental Protection Agency, the UK Department for Energy Security and Net Zero, and peer-reviewed life cycle literature (Appendix C). This approach has direct precedent in recent peer-reviewed home-care and telemedicine LCAs conducted under ISO 14040/44 and is consistent with the methodologies documented across 76 hospital LCA studies in the most recent state-of-the-science review [29,38,39]. Several of the factors applied—including those for plastics waste and home laundry water use—were derived from data sets outside the U.S. healthcare sector and may not perfectly represent device-, supplier-, or region-specific impacts. As previously stated, despite these constraints, the magnitude and directionality of our findings align with the state-of-the-science review of hospital carbon footprints, which identified travel, facilities, and consumables as the principal contributors to carbon footprint [38]. Additionally, our per-term transport savings (~234 kgCO_2_e) fall within the 0.70–372 kgCO_2_e per-consultation range reported across telemedicine LCAs [39]. System boundaries in any LCA are inherently subjective and shaped by feasibility; we set boundaries based on standard care patterns, and individual infants whose care deviated from those patterns may not be fully represented. Future analyses applying alternative emission-factor sources, including supplier-specific data and regional U.S. grid-specific electricity factors, will be valuable in further refining the environmental case for hospital-at-home models in pediatric and neonatal care.

Additionally, there is a limitation in our assessment of solid waste generation. We focused our assessment on one output metric (kgCO_2_e) in hopes of creating a framework for analysis that could be easily extrapolated to other healthcare services and pathways. However, this may have oversimplified our interpretation of waste generation. Solid waste in this analysis was characterized by item type and weight rather than by regulated waste stream (regulated medical waste, sharps, municipal solid waste), and disposal pathway emissions (incineration, autoclave sterilization, landfill) were not modeled separately. Hospital waste streams have distinct end-of-life carbon intensities, and future LCAs would benefit from stream-stratified inventory paired with facility-specific disposal-pathway emission factors.

Finally, the overall rigor of our analyses may have been influenced by two overarching components. First, the project was completed within a pre-defined engineering course at our academic center. Secondly, these analyses were the first of their kind conducted at our healthcare facility. Given this context, there were inherent limitations on time and expertise, with engineering students performing the majority of the analysis while under expert supervision. Although this framework introduced limitations, the structure of this educational course was also the catalyst for the development of our interdisciplinary team, therefore creating the opportunity to actually complete these LCAs. In spurring interdisciplinary collaboration at our facility, these initial limitations may have built the groundwork for future, and improved, analyses.

## 5. Conclusions

Given that the healthcare sector is a significant contributor to carbon emissions, there is a professional obligation across the healthcare sector to increase its sustainability efforts. This analysis demonstrated that through the creation of an interdisciplinary team, it is possible for healthcare facilities to more fully assess the environmental impact of their care practices. This LCA specifically analyzed and compared the carbon footprint of a novel care-at-home program for neonates versus standard care in the NICU. More than four-fold the carbon emissions were associated with standard care in the NICU compared to the supported home program. Transportation and solid waste accounted for the majority of the discrepancy between the two care models. These findings support continued research and development in remote-care models and/or care pathways that seek to deescalate levels of care. Our findings also support the evaluation and implementation of waste-reduction strategies in the healthcare setting. Ultimately, this analysis highlights the need for continued investment in sustainability research and initiatives across the healthcare sector. A more comprehensive understanding of the environmental impact of healthcare processes can lead to decreased waste and carbon emissions, creating cleaner and safer healthcare delivery.

## Figures and Tables

**Table 2:** Impact assessment for plastic syringes used in the care of a newborn in the NICU.

Item and Usage	Weight per Item (kg)	Usage per Day (Number of Items)	Weight per Day (kg)	Conversion Factor (kgCO_2_/kg)	Emissions per Day (kgCO_2_e)	Emissions per Term (kgCO_2_e)
**Syringe**	0.025					
High	0.025	10	0.25	3.36	0.84	11.76
Low	0.025	8	0.2	3.36	0.67	9.41
Average	0.025	9	0.225	3.36	0.76	10.58

**Table 3:** Comparison of total emissions (kgCO_2_e) for the NICU setting vs. the HGaH program.

Setting	Impact Category	Emissions (kg CO_2_e per Term)	Percentage
**NICU**			
	Energy	18.91	5.59%
	Solid waste	26.97	7.97%
	Water	0.31	0.09%
	Transportation	292.00	86.34%
	**Total**	**338.19**	
**HGaH**			
	Energy	16.34	21.18%
	Solid waste	1.74	2.26%
	Water	0.66	0.86%
	Transportation	58.4	75.71%
	**Total**	**77.14**	

## Data Availability

Data for this project have been included in the appendices and references, further inquiries can be directed to the corresponding authors.

## Abbreviations

The following abbreviations are used in this manuscript:

DHMC	Dartmouth Hitchcock Medical Center
NICU	Neonatal Intensive Care Unit
LCA	Life Cycle Assessment
HGaH	Hope Grows at Home
ISO	International Organization of Standardization

## Appendix A. NICU Nurse Interview Document

The purpose of this document is for either team members or sponsors of the LCA Project to have a conversation with nurses who work in the DHMC Intensive Care Nursery (ICN) to gather general information about caring for babies in the ICN to further improve the analysis of the environmental impact of both the ICN and the Hope Grows at Home program. Though we are looking for quantitative data, these questions and answers are more free-form and broad, allowing for a longer conversation or explanation behind them, which the team can then use to refine their environmental analysis. Furthermore, if some of these questions bring up other questions or desired information, we encourage a longer conversation to gather as much information as possible.

1. Please walk us through your routine for caring for one individual baby that is eligible/close to eligible for the Hope Grows at Home program during a single shift. Who else is involved in that routine? What supplies do you use? How are those supplies used, and what happens to them following usage (disposal, cleaning, nothing)?
2. To your knowledge, how often do parents visit the hospital while their child is in the ICN? What are the reasons that these visits occur?
3. How many babies do you care for during a single shift? How much of the time is spent on the babies that are eligible/close to eligible for the Hope Grows at Home program?
4. How much of your routine is dictated by a specific set of instructions by the hospital? How much deviation is there (qualitatively)? What are common deviations among the care for otherwise similar babies? What about less common deviations?

## Appendix B. Detailed Inventory Analysis and Impact Assessment by Impact Category

1. **Solid Waste**

**Table A1:** Impact assessment of solid waste in the NICU.

Item	Weight per Item (kg)	Usage per Day (Number of Items)	Weight per Day (kg)	Conversion Factor (kgCO_2_/kg)	Emissions per Day (kgCO_2_e)	Emissions per Term (kgCO_2_e)
**Paper towels**	0.002					
High	0.002	50	0.1	0.027	0.003	0.038
Low	0.002	20	0.04	0.027	0.001	0.015
Average	0.002	35	0.07	0.027	0.002	0.026
**Syringe**	0.025					
High	0.025	10	0.25	3.36	0.840	11.760
Low	0.025	8	0.2	3.36	0.672	9.408
Average	0.025	9	0.225	3.36	0.756	10.584
**Formula bottle**	0.025					
High	0.025	10	0.25	3.36	0.840	11.760
Low	0.025	8	0.2	3.36	0.672	9.408
Average	0.025	9	0.225	3.36	0.756	10.584
**Wipes**	0.001					
High	0.001	40	0.04	1.2	0.048	0.672
Low	0.001	25	0.025	1.2	0.030	0.420
Average	0.001	32.5	0.0325	1.2	0.039	0.546
**Diapers**	0.03					
High	0.03	10	0.3	0.13	0.039	0.546
Low	0.03	7	0.21	0.13	0.027	0.382
Average	0.03	8.5	0.255	0.13	0.033	0.464
**Gloves**	0.007					
High	0.007	20	0.14	0.026	0.004	0.051
Low	0.007	16	0.112	0.026	0.003	0.041
Average	0.007	18	0.126	0.026	0.003	0.046
**Masks**	0.004					
High	0.004	15	0.06	0.022	0.001	0.018
Low	0.004	8	0.032	0.022	0.001	0.010
Average	0.004	11.5	0.046	0.022	0.001	0.014
**Temperature probe**	0.005					
High	0.005	10	0.05	3.36	0.168	2.352
Low	0.005	6	0.03	3.36	0.101	1.411
Average	0.005	8	0.04	3.36	0.134	1.882
**Milk warmer liner**	0.04					
High	0.04	2	0.08	3.36	0.269	3.763
Low	0.04	1	0.04	3.36	0.134	1.882
Average	0.04	1.5	0.06	3.36	0.202	2.822

Table A1: Demonstrates the calculations used to determine the impact of solid waste in NICU care. Conversion factor sources can be found in Appendix C. Quantities of materials used were determined by NICU nurse interviews and care observations and assumption was made that the number of cares performed at home per day (8×/day).

**Table A2:** Impact assessment of solid waste in the HGaH.

Item	Weight per Item (kg)	Usage per Day (Number of Items)	Weight per Day (kg)	Conversion Factor (kgCO_2_/kg)	Emissions per Day (kgCO_2_e)	Emissions per Term (kgCO_2_e)
**Paper towels**	0.002					
High	0.002	50	0.1	0.027	0.003	0.038
Low	0.002	25	0.05	0.027	0.001	0.019
Average	0.002	37.5	0.075	0.027	0.002	0.028
**Syringe**	0.025					
High	0.025	0.5	0.0125	3.36	0.042	0.588
Low	0.025	0.36	0.009	3.36	0.030	0.423
Average	0.025	0.43	0.01075	3.36	0.036	0.506
**Wipes**	0.001					
High	0.001	30	0.03	1.2	0.036	0.504
Low	0.001	20	0.02	1.2	0.024	0.336
Average	0.001	25	0.025	1.2	0.030	0.420
**Diapers**	0.03					
High	0.03	8	0.24	0.13	0.031	0.437
Low	0.03	6	0.18	0.13	0.023	0.328
Average	0.03	7	0.21	0.13	0.027	0.382
**Milk warmer liner**	0.04					
High	0.04	0.29	0.0116	3.36	0.039	0.546
Low	0.04	0.14	0.0056	3.36	0.019	0.263
Average	0.04	0.215	0.0086	3.36	0.029	0.405

Table A2: Demonstrates the calculations used to determine the impact of solid waste in HGaH care. Conversion factor sources can be found in Appendix C. Quantities of materials used were determined by the number of supplies provided in the HGaH kit and assumptions around the number of cares performed at home per day (8×/day). Additionally, reusable items, such as syringes, were included in the impact assessment, but their quantities were determined based on the numbers needed during the entire 14-day course (e.g., 0.5 syringes used per day equates to 7 syringes used over the entire term of the program).

**Table A3:** Total emissions for the solid waste impact category for NICU care and the HGaH Program.

	**(a) Total Emissions (kgCO_2_e) for Solid Waste in the NICU**
High	30.96
Low	22.98
Average	26.97
	**(b) Total Emissions (kgCO_2_e) for Solid Waste in HGaH**
High	2.11
Low	1.37
Average	1.74

Table A3: Total emissions for solid waste were calculated by summing the emissions for each solid waste item.

2. **Energy**

**Table A4:** Energy inventory and impact assessment for the NICU.

Equipment	Device Wattage (kw)	Usage Time (hr)	Daily Energy Use (kwh)	Conversion Factor (kgCO_2_e/kwh)	Daily Emissions (kgCO_2_e)	Total Emissions (kgCO_2_e)
**Milk warmer**	0.25					
High	0.25	4	1.00	0.256	0.26	3.58
Low	0.25	1.33	0.33	0.256	0.09	1.19
Average	0.25	2.66	0.67	0.256	0.17	2.38
**MX 800 monitor**	0.19					
High	0.19	24	4.56	0.256	1.17	16.34
Low	0.19	24	4.56	0.256	1.17	16.34
Average	0.19	24	4.56	0.256	1.17	16.34
**Bedside lamp**	0.01					
High	0.01	6	0.06	0.256	0.02	0.22
Low	0.01	4	0.04	0.256	0.01	0.14
Average	0.01	5	0.05	0.256	0.01	0.18

Table A4: Demonstrates the calculations used to determine the energy impact of NICU care. There were three electric equipment devices that were identified in the NICU: Phillips Monitor MX800, Medela milk warmer, and a Trond bedside lamp. In these calculations, kilowatts (kW) were determined by consulting device manuals and energy requirements. Time used was determined via nursing interviews and observation. Kilowatts were then multiplied by time to get kilowatt hours (kWh). Conversion factor sources can be found in Appendix C.

**Table A5:** Energy inventory and impact assessment for HGaH.

Equipment	Device Wattage (kw)	Usage Time (hr)	Daily Energy Use (kwh)	Conversion Factor (kgCO_2_e/kwh)	Daily Emissions (kgCO_2_e)	Total Emissions (kgCO_2_e)
**MX 800 Monitor**	0.19					
High	0.19	24	4.56	0.256	1.17	16.34
Low	0.19	24	4.56	0.256	1.17	16.34
Average	0.19	24	4.56	0.256	1.17	16.34

Table A5: Demonstrates the calculations used to determine the energy impact of HGaH care. There was only one piece of electric equipment used in monitoring the HGaH infants, a Phillips monitor. In these calculations, kilowatts (kW) were determined by consulting device manuals and energy requirements. Time used was determined via the program guideline on 24 h monitoring. Kilowatts were then multiplied by time to get kilowatt hours (kWh). Conversion factor sources can be found in Appendix C.

**Table A6:** Total emissions for the energy impact category for NICU care and the HGaH Program.

	**(a) Total Emissions (kgCO_2_e) for Energy in the NICU**
High	20.14
Low	17.68
Average	18.91
	**(b) Total Emissions (kgCO_2_e) for Energy in HGaH**
High	16.34
Low	16.34
Average	16.34

Table A6: Total emissions for energy were calculated by summing the emissions for each energy item.

3. **Water**

**Table A7:** Water inventory and impact assessment for the NICU.

Activity	Usage per Performance (L)	Performances per Day	Usage per Day (L)	Conversion Factor (kgCO_2_/L)	Emissions per Day (kgCO_2_e)	Emissions per Term (kgCO_2_e)
**Handwashing (healthcare worker)**						
High	2.10	24	50.4	0.00046	0.0232	0.325
Low	2.10	12	25.2	0.00046	0.0116	0.162
Average	2.10	18	37.8	0.00046	0.0174	0.243
**Handwashing (visitor)**						
High	1.86	6	11.16	0.00046	0.0051	0.072
Low	1.86	2	3.72	0.00046	0.0017	0.024
Average	1.86	4	7.44	0.00046	0.0034	0.048
**Formula**						
High	0.15	8	1.2	0.00046	0.0006	0.008
Low	0.15	6	0.9	0.00046	0.0004	0.006
Average	0.15	7	1.05	0.00046	0.0005	0.007
**Bathing**						
High	0.84	1	0.84	0.00046	0.0004	0.005
Low	0.84	0.5	0.42	0.00046	0.0002	0.003
Average	0.84	0.75	0.63	0.00046	0.0003	0.004
**Bottle washing**						
High	0.13	8	1.04	0.00046	0.0005	0.007
Low	0.13	6	0.78	0.00046	0.0004	0.005
Average	0.13	7	0.91	0.00046	0.0004	0.006

Table A7: Demonstrates the inventory and calculations used for the impact of water in NICU care. As there are robust existing data on the water usage of common practices, water amounts per performance were determined via literature review [47,48,49,50,51]. Performances per day were obtained by nurse interviews and direct observation. Usage per day was calculated by multiplying usage (L) per activity by usage per day (e.g., 2.1 L/performance × 24 performances/day = 50.4 L/day). Emissions per day were then calculated by multiplying the usage per day by the conversion factor (Appendix C).

**Table A8:** Water inventory and impact assessment in HGaH.

Activity	Usage per Performance (L)	Performances per Day	Usage per Day (L)	Conversion Factor (kgCO_2_/L)	Emissions per Day (kgCO_2_e)	Emissions per Term (kgCO_2_e)
**Hand washing (visitor)**						
High	1.86	8	14.88	0.00046	0.0068	0.096
Low	1.86	6	11.16	0.00046	0.0051	0.072
Average	1.86	7	13.02	0.00046	0.0060	0.084
**Formula**						
High	0.15	8	1.2	0.00046	0.0006	0.008
Low	0.15	6	0.9	0.00046	0.0004	0.006
Average	0.15	7	1.05	0.00046	0.0005	0.007
**Bathing**						
High	0.84	1	0.84	0.00046	0.0004	0.005
Low	0.84	0.33	0.2772	0.00046	0.0001	0.002
Average	0.84	0.67	0.5628	0.00046	0.0003	0.004
**Bottle washing**						
High	0.13	8	1.04	0.00046	0.0005	0.007
Low	0.13	6	0.78	0.00046	0.0004	0.005
Average	0.13	7	0.91	0.00046	0.0004	0.006
**Laundry**						
High	144	1	144	0.00046	0.0662	0.927
Low	144	0.2	28.8	0.00046	0.0132	0.185
Average	144	0.6	86.4	0.00046	0.0397	0.556

Table A8: This table represents the impact assessment for water usage in the HGaH program. As there are robust existing data on the water usage of common practices, water amounts per performance were determined via literature review and product specifications [47,48,49,50,51]. Performances per day were determined via assumptions around standard home-care practices. Usage per day was calculated by multiplying usage (L) per activity by usage per day (e.g., 2.1 L/performance × 24 performances/day = 50.4 L/day). Emissions per day were then calculated by multiplying the usage per day by the conversion factor (Appendix C). One notable difference between HGaH and NICU water usage is the inclusion of a laundry category. Estimations of water usage for laundry in home settings are relatively well established. Laundry in our NICU is performed offsite from the hospital, and fell outside the scope of our LCA.

**Table A9:** Total emissions for the energy impact category for NICU care and the HGaH program.

	**(a) Total emissions (kgCO_2_e) for water in the NICU**
High	0.416
Low	0.200
Average	0.308
	**(b) Total emissions (kgCO_2_e) for water in HGaH**
High	1.043
Low	0.270
Average	0.657

Table A9: Total emissions for water were calculated by summing the emissions for each activity requiring use of water.

4. **Transportation**

**Table A10:** Transportation impact assessment for NICU care.

Transport Mode	Round Trip Distance (mi)	Visits per Term	Conversion Factor (kgCO_2_/mi)	Emissions per Trip (kgCO_2_e)	Emissions per Term High (kgCO_2_e)	Emissions per Term Low (kgCO_2_e)	Emissions per Term Average (kgCO_2_e)
**Passenger vehicle**							
High	142	14	0.4	56.40	789.60	338.4	564
Low	5	6	0.4	2.00	28.00	12	20
Average	73	10	0.4	29.20	408.80	175.2	**292**

Table A10: Demonstrates the inventory and calculations used to determine the impact of transportation in NICU care. To determine the distance traveled, we provided a high and low estimation of distance based on assumptions about hospital location relative to patients’ homes. For the low travel distance estimation, we assumed a round-trip distance of 5 miles, as almost all patients at our healthcare system live more than 2.5 miles from the hospital. We used a high estimation of 142 miles round-trip. The nearest Level III NICU is 71 miles from our facility, so we assumed that preterm newborns would need to travel this distance in order to receive equivalent care. The number of visits for NICU care was determined by reviewing NICU visitor log books. We used the EPA’s conversion factor for a standard passenger vehicle to determine our emissions (Appendix C). Our emissions/trip variable was then multiplied by the number of visits per term to obtain a total emissions amount per term.

**Table A11:** Transportation impact assessment for HGAH.

Transport Mode	Round Trip Distance (mi)	Visits Per Term	Conversion Factor (kgCO_2_/mi)	Emissions per Trip (kgCO_2_e)	Emissions per Term High (kgCO_2_e)	Emissions per Term Low (kgCO_2_e)	Emissions per Term Average (kgCO_2_e)
**Passenger vehicle**							
High	142	2	0.4	56.40	112.80	112.8	112.8
Low	5	2	0.4	2.00	4.00	4	4
Average	73	2	0.4	29.20	58.40	58.4	**58.4**

Table A11: Demonstrates the inventory and calculations used to determine the impact of transportation in NICU care. The assumptions made about distance traveled were the same as in Table A10. The HGaH program has an established practice of conducting 2 in-person appointments during the first two weeks of the program. We used the EPA’s conversion factor for a standard passenger vehicle to determine our emissions (Appendix C). Our emissions/trip variable was then multiplied by the number of visits per term to obtain a total emissions amount per term.

## Appendix C

**Table A12:** Impact assessment conversion factors.

Conversion Factors			
Impact Category	Inventory Item	Conversion Factor Used	References
**Solid Waste**			
	Paper towels	0.27 kgCO_2_/kg	[14]
	Syringe	3.36 kgCO_2_/kg	[30]
	Temperature probe	3.36 kgCO_2_/kg	[30]
	Milk warmer liner	3.36 kgCO_2_/kg	[30]
	Formula bottle	3.36 kgCO_2_/kg	[30]
	Wipes	1.2 kgCO_2_/kg	[31]
	Diapers	0.13 kgCO_2_/kg	[32]
	Gloves	0.026 kgCO_2_/kg	[33]
	Masks	0.022 kgCO_2_/kg	[34]
**Energy**			
	Milk warmer	0.256 kgCO_2_/kwh	[35]
	MX 800 vitals monitor	0.256 kgCO_2_/kwh	[35]
	Bedside lamp	0.256 kgCO_2_/kwh	[35]
**Water**			
	Handwashing	0.00046 kgCO_2_/liter	[36]
	Formula	0.00046 kgCO_2_/liter	[36]
	Bathing	0.00046 kgCO_2_/liter	[36]
	Bottle washing	0.00046 kgCO_2_/liter	[36]
	Laundry (home)	0.00046 kgCO_2_/liter	[36]
**Transportation**			
	Passenger vehicle	0.4 kgCO_2_/mile	[37]
